# Wool-Growers

**Published:** 1869-11

**Authors:** 


### WOOL-GROWERS.

We are requested, by one of our western wool-growers, to publish in the Journal a list of the best sheep-shearing machines, where they may be purchased, the prices, and, in fact, such information as we may be able to gather respecting that branch of invention.

We are not aware that there are any such machines in use to any extent, though there are several patented; and we would be glad if some of our readers could furnish us with a brief description of a practical machine for this important branch of industry.

The demand for such an invention must be very great, when we refer to the wool crop of 1868, amounting to about 177,000,000 pounds in the United States. We hope to be able to supply the desired information in our next issue.

